# Supplementary figures and images for: Investigating DNA methylation as a potential mediator between pigmentation genes, pigmentary traits and skin cancer
Source: Pigment Cell Melanoma Res. 2020 Dec 10;34(5):892–904. doi: 10.1111/pcmr.12948 (PMC8518056; doi:10.1111/pcmr.12948)

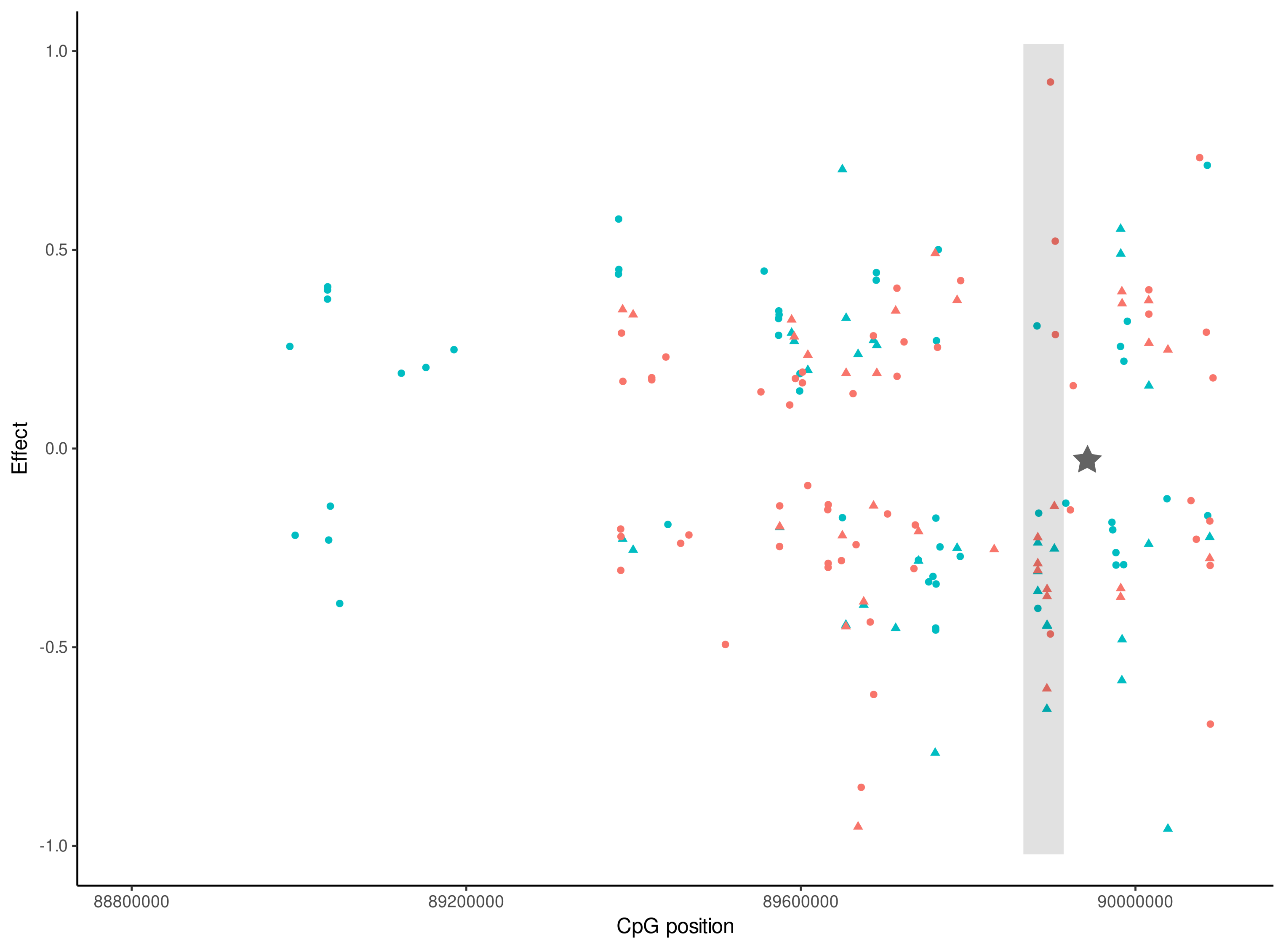

Supplement: Supplementary file 1 — Figure S1 [file PCMR-34-892-s003.png]
